# The Association Between Serum Zinc Levels and Anthropometric Measurements and Nutritional Indicators in Children With Idiopathic Short Stature

**DOI:** 10.7759/cureus.24906

**Published:** 2022-05-11

**Authors:** Daisuke Sugawara, Eishi Makita, Misa Matsuura, Ko Ichihashi

**Affiliations:** 1 Pediatrics, Saitama Medical Center Jichi Medical University, Saitama, JPN

**Keywords:** idiopathic short stature, insulin-like growth factor-1, growth disorders, body mass index, zinc

## Abstract

Background

Zinc (Zn) is an essential trace element, and its deficiency causes various symptoms, such as anemia, short stature, and poor weight gain, in children. Several studies have reported an association between Zn deficiency and short stature in children. However, few studies have reported on the relationship between serum Zn levels, body mass index (BMI), and nutritional indicators such as albumin, amino acids, and vitamin D.

Methods

We retrospectively analyzed the data of 56 children with idiopathic short stature. We investigated the mean serum Zn levels and the relationships among serum Zn levels and height standard deviation score (SDS), bodyweight SDS, BMI SDS, hemoglobin (Hb), albumin, alkaline phosphatase (ALP), insulin-like growth factor-1 (IGF-1), 25-hydroxyvitamin D (25(OH)D), and amino acid levels.

Results

The mean serum Zn levels of the study participants were 70.3±10 µg/dL. Serum Zn levels correlated significantly with weight SDS (r=0.472, p<0.001) and BMI SDS (r=0.416, p<0.001). In contrast, no significant association was found between serum Zn levels and height SDS (r=0.217, p=0.078). We found significant positive correlations between serum Zn levels and Hb and IGF-1 SDS (Hb: r=0.333, p=0.012; IGF-1 SDS: r=0.372, p=0.00478). Serum Zn levels were not correlated with albumin, ALP, 25(OH)D, and amino acid levels.

Conclusions

Serum Zn levels are associated with anthropometric measurements, especially body weight and BMI. It is important to evaluate Zn levels in children who fail to thrive, even in developed countries.

## Introduction

Zinc (Zn) is an essential trace element that plays an important role in enzyme and protein activity and is a structural component of cells in humans. Zn is abundant in eggs, nuts, and animal proteins such as meat and seafood. Low intake of these foods due to allergies, unbalanced diets, or economic reasons is a risk factor for Zn deficiency, resulting in various symptoms such as anemia, dermatitis, loss of appetite, dysgeusia, and impaired immunity [1-4]. In children with Zn deficiency, short stature and poor weight gain have also been reported [5].

Several studies have reported an association between Zn deficiency and short stature in children and that Zn deficiency is present in a certain proportion of short-statured children [6-8]. However, there are only a few reports on the relationship between serum Zn levels and body mass index (BMI) and nutritional indicators such as albumin, amino acids, and vitamin D [9].

Here, we report the mean serum Zn levels and the association between serum Zn levels and anthropometric measurements and laboratory results, including nutritional indicators, in short-statured children.

## Materials and methods

This retrospective study was conducted by reviewing patients’ electronic medical records. We included 63 prepubertal, short-statured Japanese children who visited our facility between January 2018 and December 2020. Short stature is defined as a height standard deviation score (SDS) of less than -2. Height SDS was calculated for sex- and age-matched normal Japanese children [10]. Anterior pituitary hormone levels were measured in all patients, and growth hormone (GH) provocation tests were conducted. GH deficiency (GHD) was defined as peak GH levels below 6.0 ng/mL according to the criteria of the Japanese Society for Pediatric Endocrinology [11]. Six patients had GH deficiency. All girls underwent a chromosome examination using the G-banding method. One patient had Turner syndrome. None of the patients had hypopituitarism and congenital disorders, except for GHD and Turner syndrome, respectively. The final study sample comprised 56 patients with idiopathic short stature.

We retrieved data on age, sex, height, body weight, BMI, medical history, and laboratory assays. Height and body weight measurement and laboratory assays were measured during the patients’ first visit to our hospital for short stature. BMI was calculated as weight in kilograms divided by the square of the height in meters. Laboratory assays were performed for hemoglobin, albumin, alkaline phosphatase (ALP), Zn, insulin-like growth factor-1 (IGF-1), 25-hydroxyvitamin D (25(OH)D), and amino acids (histidine, methionine, and glutamine). We investigated histidine, methionine, and glutamine, as these amino acids promote the absorption of Zn in animal proteins [12]. Serum Zn levels and 25(OH)D levels were measured using a colorimetric assay (SRL Co., Ltd., Tokyo, Japan) and a chemiluminescent enzyme immunoassay (SRL Co., Ltd.), respectively. Amino acid analysis was performed using liquid chromatography-mass spectrometry (SRL Co., Ltd.). We investigated the mean serum Zn levels in patients with idiopathic short stature. We also investigated the relationships between serum Zn levels and height SDS, bodyweight SDS, BMI SDS, and laboratory assays (hemoglobin, albumin, ALP, IGF-1, 25(OH)D, histidine, methionine, and glutamine). BMI SDS and IGF-1 SDS were calculated for sex- and age-matched normal Japanese children [10,13]. Anemia was diagnosed according to the World Health Organization definition [14].

This study was conducted in accordance with the principles of the Declaration of Helsinki and was approved by the institutional ethics review board of our facility (approval no. S20-073, dated October 5, 2020).

Statistical analysis

Data are presented as mean ± standard deviation (SD). We performed the Mann-Whitney U test to determine possible differences between the two groups. The relationships between serum Zn levels and other factors were investigated using Pearson’s correlation coefficient test. All statistical analyses were performed using EZR version 1.54 software (Saitama Medical Center, Jichi Medical University, Saitama, Japan) [15]. Statistical significance was set at p<0.05.

## Results

Characteristics of study participants

The characteristics of the study participants are listed in Table 1. We included 56 children with idiopathic short stature, of which 30 (53.5%) were boys. The mean serum Zn level was 70.3±10 µg/dL. Serum Zn levels were not significantly different between boys and girls (71.52±11.02 vs 68.88±8.86 µg/dL, p=0.331). Serum Zn levels in nine children were less than 60 µg/dL. The mean hemoglobin level was 12.79±0.78 g/dL. Four (5.2%) children had anemia, according to the World Health Organization criteria. The mean IGF-1 SDS was -1.36±0.87 ng/dL. None of the participants had severe anemia or malnutrition.

**Table 1:** Background data of study participants included in the analysis Results are expressed as mean± SD and range SD: standard deviation, SDS: standard deviation score, BMI: body mass index, Zn: zinc, ALP: alkaline phosphatase, IGF-1:insulin growth factor-1, 25OHD: 25-hydroxyvitamin D

	Mean±SD	Range
Age (years)	6.0±2.5	2.25-10.45
Height SDS	-2.34±0.32	-2.01-3.1
Weight SDS	-1.64±0.85	-2.95-+0.11
BMI SDS	-0.07±0.94	-2.24-+1.86
Zn (µg/dL)	70.3±10.0	56-96
Hemoglobin (g/dL)	12.79±0.78	10.8-14.5
Albumin (g/dL)	4.55±0.28	3.8-5.2
ALP (IU/L)	694.4±141.2	363-944
IGF-1 (ng/ml)	81.94±37.74	32-211
IGF-1 SDS	-1.36±0.87	-3.52-+0.81
25OHD (ng/mL)	23.49±6.09	10-40.8
Histidine (nmol/mL)	77.24±13.64	55.9-109
Methionine (nmol/mL)	25.36±7.96	11.7-49.5
Glutamine (nmol/mL)	538.2±76	321-664.9

Correlations between serum Zn levels and anthropometric measurements

**Figure 1:**
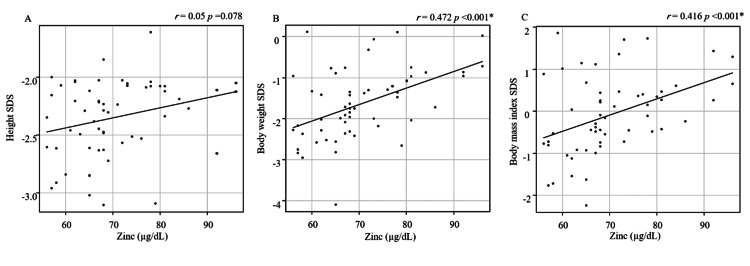
Correlations between serum zinc levels and height standard deviation score (SDS), bodyweight SDS, and body mass index SDS A: height SDS; B: bodyweight SDS; C: body mass index SDS *p<0.05 is indicative of statistical significance.

We investigated the association between height SDS, weight SDS, BMI SDS, and serum Zn levels (Figure 1). Serum Zn levels were moderately correlated with weight SDS (r=0.472, p<0.001) and BMI SDS (r=0.416, p<0.001). In contrast, no significant association was found between serum Zn levels and height SDS (r=0.217, p=0.078).

Correlations between serum Zn levels and laboratory results

**Figure 2:**
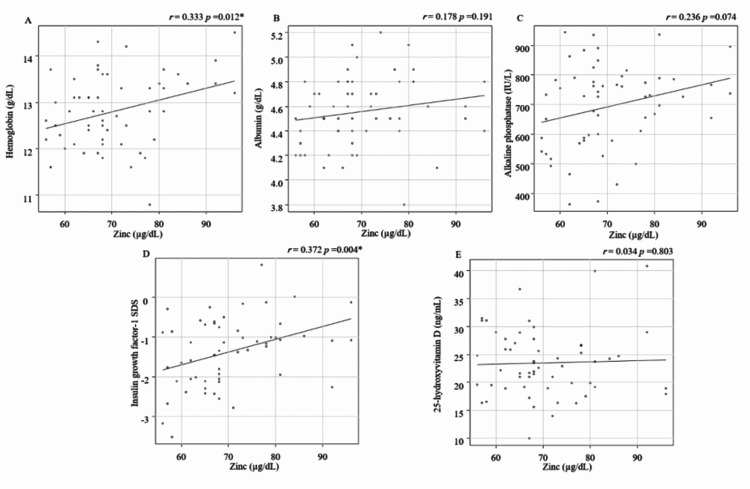
Correlations between serum zinc levels and hemoglobin, albumin, alkaline phosphatase, insulin growth factor-1, 25-hydroxyvitamin D A: hemoglobin, B: albumin; C: alkaline phosphatase; D: insulin-like growth factor-1; E: 25-hydroxyvitamin D) *p<0.05 is indicative of statistical significance.

Additionally, we investigated the association between hemoglobin, albumin, ALP, IGF-1 SDS, 25(OH)D, histidine, methionine, glutamine, and serum Zn levels. Moderate positive correlations were found between serum Zn levels and Hb and IGF-1 SDS (Hb: r=0.333, p=0.012; IGF-1 SDS: r=0.372, p=0.00478; Figure 2).

**Figure 3:**
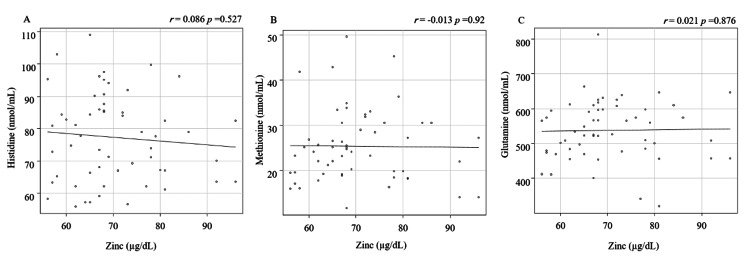
Correlations between serum Zn levels and histidine, methionine, and glutamine A: histidine; B: methionine; C: glutamine

We observed no significant associations between serum Zn levels and albumin (r=0.178, p=0.191), ALP (r=0.236, p=0.074), or 25(OH)D (r=0.034, p=0.803). Serum Zn levels were not significantly correlated with histidine, methionine, and glutamine levels, which promote Zn absorption (Figure 3; histidine: r=-0.0862, p=0.527; methionine: r=-0.013, p=0.92; glutamine: r=0.021, p=0.876).

## Discussion

In this study, we measured serum Zn levels and their association with anthropometric measurements and laboratory results, including nutritional indicators, in children with idiopathic short stature. The results of this study revealed three clinical findings. First, the mean serum Zn levels were 70.3±10 µg/dL, and nine (16.0%) children had serum Zn levels below 60 µg/dL. Zn deficiency was noted in children with idiopathic short stature at a fixed rate. Second, serum Zn levels did not correlate with height SDS but were moderately correlated with weight SDS and BMI SDS in children with idiopathic short stature. Third, serum zinc levels were moderately correlated with Hb and IGF-1 SDS in children with idiopathic short stature.

There are several reports on the frequency of Zn deficiency in children with short stature. Kaji et al. reported that 36% of children with short stature had Zn deficiency defined as less than 70 µg/dL [16]. Additionally, Hamza et al. reported significantly lower serum Zn levels in children with short stature than in children without short stature [7]. Similarly, our results show low serum Zn levels in a certain proportion of children with short stature.

Regarding the association between serum Zn levels and anthropometric measurements, our results showed that serum Zn levels were not correlated with height SDS but were moderately correlated with weight SDS and BMI SDS in children with idiopathic short stature. Zn deficiency is associated with short stature, and its pathophysiology is thought to include decreased GH secretion and decreased GH receptors in the liver [7,16]. However, the mechanism by which Zn deficiency affects short stature is unclear. There are many causes of short stature in children, such as GHD, familial short stature, and hypothyroidism. Therefore, although we observed Zn deficiency in a certain proportion of children with short stature, it was not correlated with height SDS. On the contrary, bodyweight SDS and BMI SDS showed a slightly positive correlation with serum Zn levels. Zn deficiency reduces appetite by inhibiting the release of neuropeptide Y from the hypothalamus [17]. Loss of appetite due to Zn deficiency can affect the body weight and BMI, which reflect oral intake/appetite. Therefore, it is possible that serum Zn levels are moderately correlated with body weight and BMI, as seen in our results.

Laboratory test results showed that serum Zn levels were moderately correlated with Hb and IGF-1 SDS in children with idiopathic short stature. Zn deficiency causes anemia because the Zn finger protein GATA-1 is involved in the differentiation and proliferation of erythroblasts [2]. Zn deficiency, therefore, impairs erythroblast differentiation and proliferation, resulting in anemia [2]. However, participants in the present study had a low weight SDS possibly because of anemia due to nutritional deficiencies other than Zn.

Regarding the relationship between Zn deficiency and IGF-1, Zn deficiency decreases circulating IGF-1 levels independent of the total energy intake [18]. A possibility regarding the effect of Zn deficiency on IGF-1 levels maybe that Zn is involved in the expression of GH receptor and GH binding protein in the liver, and its deficiency reduces their expression, leading to low IGF-1 levels [19].

This study has several limitations. First, we measured serum Zn levels during afternoon fasting. Serum Zn levels are known to have diurnal fluctuations and are lower in the afternoon than in the morning [20]. Therefore, we may have overestimated Zn deficiency in this study. However, we did not examine the numerical values but the correlation between serum Zn levels and each analyte; thus, our results may still be significant because the time of measurement may have had little impact on the study results. Second, we conducted a retrospective study at a single institution, with small sample size. Therefore, the results of this study may not reflect the trend in the general population. Third, although we confirmed no extreme decline in food intake (through interviewing), we did not enquire about detailed food intake such as calories, carbohydrates, proteins, fats, and trace elements. It cannot be denied that anthropometric measurements, IGF-1, and Hb may be affected by the amount of food consumed.

## Conclusions

In conclusion, Zn deficiency, though traditionally considered common in developing countries, was present in a certain proportion of the population, even in a developed country such as Japan. Our data suggest that serum Zn levels are associated with anthropometric measurements, especially body weight and BMI. It is therefore important to evaluate Zn in children who fail to thrive, even in developed countries. In the future, we plan to administer Zn to children with Zn deficiency and investigate whether their growth rate can improve.
